# Nanobiotechnology: A New Frontier for Brain Disorders

**DOI:** 10.3390/ijms23179603

**Published:** 2022-08-24

**Authors:** Pasquale Picone

**Affiliations:** 1 Istituto per la Ricerca e l’Innovazione Biomedica, CNR, Via U. La Malfa 153, 90146 Palermo, Italy; pasquale.picone@irib.cnr.it; 2 Dipartimento di Scienze e Tecnologie Biologiche Chimiche e Farmaceutiche, Università di Palermo, Viale delle Scienze, 90128 Palermo, Italy

Brain disorders, such as neurodegenerative diseases (NDs) and tumors (more than 600 pathologies), are a serious health problem, resulting in brain dysfunctions that limit normal activities, with a significant economic impact [1]. The major difficulty for the treatment of brain diseases is the presence of biological barriers (the blood–brain barrier (BBB) and blood–cerebrospinal fluid barrier) limiting brain accessibility and reducing the efficacy of therapies, with a number of side effects due to the dispersion of the drugs which fail to enter the central nervous system (CNS) [2,3]. Furthermore, it is important to administer therapeutic agents to the brain lesions present in different regions of the CNS without affecting other normal CNS tissues in order to avoid further damage. In addition, at the cellular level, it is very important that formulations may selectively target the specific brain cells (neuron, microglia, oligodendrocytes or astrocytes) involved in different brain pathologies.

In this context, the nanobiotechnology approach should offer unique opportunities to (i) improve drug bioavailability (the protection of a drug from degradation); (ii) overcome physiological barriers; (iii) enable targeted delivery and controlled release; and, consequently, (iv) reduce the doses and frequency of administration by limiting potential adverse effects. All of these advantages would allow an increase in the efficacy of therapies for the treatment of brain disorders. Several studies have focused on developing drug delivery systems (DDSs) for the brain, such as nanoparticles, dendrimers, liposomes, vesicles and nanogels [3,4,5,6,7], as a potential approach to contrast NDs.

Moreover, magnetic nanoparticles have received much attention as systems for the treatment of cerebral pathologies. In particular, they have been chosen for their ease of preparation and their advantageous properties such as composition, size, surface morphology, functionalization and ability to cross the BBB, which could be very promising for future clinical applications [8,9].

Recently, brain delivery systems capable of transporting organelles such as mitochondria have also been designed [10,11].

Extracellular vesicles (EVs), in particular, the exosomes (EXOs), have been explored as important tools for brain therapy [12,13]. Specifically, EXOs are emerging as DDSs due to their characteristics such as stability, biocompatibility, low immunogenicity (invisibility when circulating in the bloodstream), ability to overcome natural barriers and intrinsic targeting ability. EXOs are also promising for future clinical applications in the diagnosis of NDs and are being studied for their usefulness in detecting and predicting disease prior to the emergence of symptoms [12,13]. The diagnostic potential of EXOs is due to the fact that nanovesicles have a specific biomolecular profile represented by proteins, nucleic acids and lipids that can form a “fingerprint” of the mother cells, and therefore reflect pathological conditions when cellular changes occur. In addition, EXOs are readily present in almost all body fluids (blood, urine, breast milk, saliva, sperm, amniotic fluid, cerebrospinal fluid (CSF) and lymph) [12,13].

Recent work on EXOs has pointed out various aspects related to their roles in the pathogenesis, diagnosis and treatment of Alzheimer’s disease (AD) [14]. EXOs can carry different cargoes, ranging from drugs such as quercetin and curcumin to enzymes such as neprilysin and insulin-degrading enzyme, as well as miRNA [14], for the purpose of counteracting AD. Moreover, EXOs could act as Aβ scavengers, i.e., one of the main actors involved in AD [15]. In fact, through the molecules present on the surface (glycans on glycosphingolipids), the EXOs might bind to Aβ and play an important role in Aβ brain clearance [14].

The mechanisms by which EVs regulate the neuro-inflammation processes of the microglia remain largely unexplored. Recently, the action of EVs derived from human exfoliated deciduous teeth stem cells (SHEDs) (EVs-SHEDs) on human microglial cells has been investigated [16]. In particular, EVs-SHEDs encourage a rapid increase in the intracellular Ca2+ level, and promote ATP production and microglial migration. The results demonstrate that EVs-SHEDs have an immunomodulatory effect and induce microglial motility through P2X4R/MFG-E8-dependent mechanisms [16].

Nanotechnologies have been used for the development of new therapies for multiple sclerosis (MS) [17]. In particular, natural and artificial vesicles and nanoparticles have been proposed as valid nanotherapeutic approaches. In this context, nanovectors have been investigated for use as DDSs and as vectors for antigen-specific immunomodulation [17] or a drug-conjugated antigen approach which combines two therapeutic strategies: antigen-specific immunotherapies and immunomodulatory agents [17].

Currently, plant virus nanoparticles (NPs) represent an innovative solution as DDSs for Shh-Dependent Medulloblastoma (MB). In particular, Tomato Bushy Stunt Virus (TBSV) nanoparticles have been proposed as an effective vehicle for the targeted delivery of chemotherapeutics to MB in order to reduce toxicity [18].

Selenium nanoparticles showed a protective effect in the neuroglial networks of the cerebral cortex in ischemia/reoxygenation conditions. Such nanoparticles suppress ischemia-induced increases in cytosolic Ca2+ and necrotic cell death by the activation of neuroprotective A2 astrocytes [19].

The set of scientific discoveries illustrated in this Special Issue highlights the great potential of nanotechnologies for the development of new therapies for CNS pathologies. In the future, greater interaction between the fields of materials science, bioengineering, biology and medicine will certainly allow a more applicative use of nanotechnology.
